# Biochar mitigates dimethylarsenate-stimulated methane emissions mediated by DOM-microbe interactions in paddy soils

**DOI:** 10.3389/fmicb.2026.1958612

**Published:** 2026-09-18

**Authors:** Yukun Kang, Lei Jin, Jiyang Du, Jinpeng Lv, Yi Tang, Jian Zhou, Jie Liu, Yuping Yang, Yuehui Jia

**Affiliations:** 1 Key Laboratory of Urban Agriculture in North China, Ministry of Agriculture and Rural Affairs, Beijing University of Agriculture, Beijing, China; 2 College of Bioscience and Resources Environment, Beijing University of Agriculture, Beijing, China; 3 Transport Planning and Research Institute, Ministry of Transport, Beijing, China

**Keywords:** biochar, dissolved organic matter, methane, methylated arsenic, soil microorganisms

## Abstract

**Introduction:**

Paddy soils represent key biogeochemical interfaces linking arsenic (As) transformation and carbon (C) cycling. Dimethylarsenate (DMAs), a widespread organoarsenic contaminant in rice paddies, can alter methane (CH_4_) dynamics, but the underlying mechanisms based on dissolved organic matter (DOM)-microbe interaction and the mitigation potential of biochar still remained insufficiently understood.

**Methods:**

Here, we investigated As biogeochemistry, CH_4_ emissions, DOM quantity and composition, microbial community assembly and functional gene variations through a 98-day microcosm incubation experiment using paddy soil amended with DMAs alone and DMAs combined with biochar.

**Results:**

We found that DMAs greatly promoted CH₄ emissions in a dose-dependent manner, with the DMAs120 treatment increasing cumulative CH₄ emissions by 60.4% relative to the control. DMAs underwent significant biotransformation, declining from 98.7% on the 7th day to 26.2% of the total As by day 98. Porewater DOC concentrations were significantly increased by up to 1.81-fold (DMAs120, day 28). PARAFAC analysis revealed that DMAs shifted DOM composition toward labile protein-like fractions, with tryptophan-like (C2) and tyrosine-like (C3) components increasing while microbial humic-like (C1) and terrestrial humic-like (C4) fractions decreased. Such labile DOM precisely matched substrate requirements of enriched anaerobic fermentative microbes, establishing synergistic metabolic networks to supply methanogenic precursors. DMAs markedly increased *mcrA* gene abundance by 4.2–7.6 times with negligible change in *pmoA*, confirming stimulated methanogenesis rather than suppressed oxidation. By contrast, biochar effectively decreased % DMAs in porewater, reducing CH_4_ emissions by 54.3% after 2% biochar amendment. Biochar also decreased DOC concentrations and protein-like DOM (C2, C3), reducing bioavailable substrates for methanogens. Furthermore, biochar suppressed *mcrA* gene abundances by 12.7–81.3%, while increased that of *pmoA* gene by 7.1–7.5 times, as well as increased porewater As(V) and enriched ammonia-oxidizing *Candidatus_Nitrosopelagicus* to potentially or indirectly stimulate anaerobic CH₄ oxidation.

**Discussion:**

These results establish mechanistic connections between DMAs biotransformation, DOM turnover and CH_4_ production in paddy soils, and verify that biochar restructures DOM-microbe interactions to mitigate DMAs-stimulated CH_4_ emissions. This study deepens insights into As-C biogeochemical coupling and offers practical support for simultaneous As remediation and greenhouse gas control in paddy ecosystems.

## 1 Introduction

Global climate change represents one of the most severe threats facing humanity in the 21st century, with methane (CH₄) playing a pivotal role in global warming as the second most important greenhouse gas behind carbon dioxide (CO_2_) (Manzanilla-Pech et al., 2022; Kim et al., 2023). Although CH₄ has a relatively short atmospheric lifetime of approximately 12 years, its 100-year global warming potential (GWP) is 28 times that of CO₂, and its 20-year GWP exceeds 80-fold, making its impact on near- to medium-term climate change particularly pronounced (Intergovernmental Panel on Climate Change [IPCC], 2021). Moreover, CH₄ emissions from paddy soils constitute a critical carbon loss pathway that can degrade soil fertility (Qian et al., 2023). Rice serves as the staple food for over half of the global population, and rice production underpins global food security. Nevertheless, the anaerobic conditions within paddy fields render them a major anthropogenic source of CH₄ emissions. Recent estimates suggested that global rice paddies emit approximately 30–40 Tg CH₄ annually, accounting for roughly 10% of total anthropogenic CH₄ emissions (Saunois et al., 2025). Over the past six decades, greenhouse gas emissions from global paddy fields have nearly doubled, largely driven by expanded cultivation areas and intensive agricultural practices such as straw incorporation (Zhang et al., 2026). Given continuous global population growth and rising rice demand, mitigating CH₄ emissions from paddy soils has become a priority for agricultural climate change mitigation.

Meanwhile, arsenic (As) contamination in paddy soils constitutes a worldwide environmental crisis, particularly severe in major rice-producing regions of Asia. Under flooded anaerobic conditions during rice cultivation, As in soil is readily mobilized and taken up by rice plants. This not only impairs rice yield and grain quality, but also poses risks to human health via food chains (Zhao et al., 2010; Zhu et al., 2014). Among diverse As species detected in paddy soils, dimethylarsenate (DMAs), a dominant organic As species, has received increasing attention in recent years. Existing studies demonstrated that DMAs is closely linked to rice “straighthead disease,” which can trigger substantial grain yield losses (Tang et al., 2020). More importantly, the production and transformation of DMAs are tightly coupled with anaerobic microbial processes in paddy soils. Current evidence revealed that methanogenic archaea could degrade DMAs through demethylation (Chen et al., 2019; Chen et al., 2023), whereas other studies have reported that methanogens also mediated the methylation of inorganic As and ultimately produce methylated As (Wang et al., 2014). Besides, it was also reported that DMAs degradation in paddy soils was closely linked to methanogenesis, for example, methylotrophic methanogenic archaea (e.g., *Methanomassiliicoccus*, *Methanosarcina*) can utilize the methyl groups of DMAs as substrates for methanogenesis to stimulate CH_4_ production (Chen et al., 2023). These complex couplings between As (de)methylation processes and CH₄ metabolism not only suggest that As contamination may exert substantial impacts on CH₄ emissions from paddy fields, but also imply an interaction between microbial DMAs degradation and CH₄ emissions through the supply of methyl carbon sources. However, research exploring how DMAs affects CH₄ emissions from paddy soils remains limited.

Soil dissolved organic matter (DOM) serves as a critical link connecting soil carbon cycling and microbial metabolism. Its concentration and composition directly determine the quantity and quality of substrates available to methanogens, making DOM a core regulator of CH₄ emissions from paddy soils (Wu et al., 2021). DOM in paddy soils has complex sources, including dissolution of native soil organic matter, plant root exudates, and straw degradation products, as well as extracellular polymeric substances and cell lysates generated during microbial metabolism (Wang et al., 2026). Studies have demonstrated a close interactive relationship between DOM composition and microbial community structure. In particular, DOM composition shapes microbial community structure and function, while microbial metabolic activities in turn modify DOM quantity and composition, with both jointly regulating soil carbon cycling processes (Wu et al., 2023). To date, most investigations have focused on how methanogenesis influenced DMAs transformation. By contrast, how exogenous DMAs reciprocally modulates CH₄ emissions and the underlying mechanisms remain poorly understood. In particular, a key unresolved scientific question concerns how the degradation and transformation of DMAs reshapes the quantity and composition of soil DOM, thereby altering DOM-microbe interactions and regulating methanogenesis.

Biochar, a carbon-rich material produced by pyrolysis of biomass under oxygen-limited conditions, has shown broad application prospects in soil improvement, pollution remediation, and greenhouse gas mitigation due to its developed pore structure, abundant surface functional groups, and good chemical stability (Kabir et al., 2023). Previous studies have demonstrated that biochar could facilitate As(V) reduction in paddy soils, yet its influences on the transformation behaviors of DMAs remain poorly clarified (Qiao et al., 2018; Yang et al., 2018). Meanwhile, biochar can also regulate soil microbial community structure and modulate the activities of methanogens and methanotrophs, thereby altering CH_4_ emissions from paddy fields (Wang et al., 2019; Somboon et al., 2024). However, most current studies about biochar effects on As contamination and CH_4_ emissions in paddy fields have been conducted independently. Few studies integrated both processes to examine whether biochar can simultaneously achieve As remediation and CH₄ mitigation. Particularly in DMAs-contaminated paddy soils, how biochar reshapes DOM-microbe interactions by regulating DOM composition and microbial communities, thereby simultaneously affecting As speciation and CH_4_ emissions, remains unclear.

Based on the aforementioned research background and scientific questions, we collected paddy soils from typical rice-producing regions of China to systematically characterize CH₄ emission dynamics under DMAs stress and explored the regulatory effects of biochar application on DMAs-stimulated CH_4_ emissions. The specific research objectives were: (1) to uncover the effects of DMAs on soil CH₄ fluxes, DOM components and microbial community structure; (2) to clarify the microscale mechanisms whereby DMAs modulates paddy soil CH₄ emissions, with an emphasis on how DMAs stress and transformation (especially degradation) alter DOM-microbe interactions; and (3) to elucidate the pathways through which biochar regulates DMAs-stimulated CH₄ emissions, by integrating multiple lines of evidence including DOM composition, microbial communities and key soil physicochemical properties. This study could advance mechanistic understanding of As-C coupled biogeochemical cycles in paddy ecosystems, and carry important theoretical implications for developing synergistic technologies to control As pollution and mitigate greenhouse gas emissions in rice paddies.

## 2 Materials and methods

### 2.1 Soil collection and biochar preparation

The topsoil (0–20 cm) was collected from a paddy field in Jiaxing City, Zhejiang Province, China (30°50′7.7”N, 120°43′5.7″E) using five-point sampling method. Arsenic concentration of the soil was 4.28 mg/kg and the pH value was 6.52. Once transported to the laboratory, the soil samples were air-dried, mixed and passed through a 2-mm sieve. The biochar was produced at 450 °C using rice straw which collected from the same region with soil samples. Arsenic concentration of the biochar was 0.74 mg/kg and the pH value was 9.98. Other physicochemical properties of the soil and biochar (BC) can refer to the previous studies (Yang et al., 2020; Cui et al., 2023).

### 2.2 Microcosm experiment design

A 98-day microcosm incubation experiment was carried out at 25 °C in dark. The experiment included five treatments, namely (1) control (without DMAs and biochar addition); (2) addition of low dose of DMAs (DMAs60), namely 0.5 times of the risk control value of soil contamination for agricultural land of China (GB 15618–2018), i.e., 60 mg As kg^−1^ for soil with pH 6.5–7.5; (3) addition of high dose of DMAs (DMAs120), namely the risk control value of soil contamination for agricultural land, i.e., 120 mg As kg^−1^ for soil with pH 6.5–7.5; (4) addition of high dose of DMAs and biochar at 1% dose (*w*/*w*) (DMAs120 + BC1); (5) addition of high dose of DMAs and biochar at 2% dose (*w*/*w*) (DMAs120 + BC2). There were four replicates for each treatment, giving a total of 20 microcosm systems. After mixing the soil with appropriate amount of DMAs (added as C_2_H_6_AsNaO_2_·3H_2_O) and biochar, 200 g of the mixture was filled into each specially designed suction flask (Li H. Q. et al., 2025). In order to simulate the natural flooding condition of paddy soil, 250 mL deionized water was added to each suction flask to keep a 3 cm water layer, and the flask’s air inlet pumped clean air at a consistent airflow rate of 0.5 L/min to refresh the air in the headspace through connecting to an air pump. A soil porewater collector was carefully inserted into the soil for porewater collection. Both air inlet and outlet were connected with a three-way stopcock for the collection of CH_4_. Samples of porewater and CH_4_ were collected on days 0, 3, 7, 14, 21, 28, 35, 42, 56, 70, 84 and 98, and soil samples were collected at the end of incubation using the same sampling methods according to our previous study (Li H. Q. et al., 2025). All samples were stored at −20 °C or −80 °C depending on the various analytical needs.

### 2.3 Determination of As concentration, As species and major physicochemical properties in porewater

After being acidified with 2% HNO_3_ and passed through a 0.22-μm filter, the total As concentrations and species in porewater were determined by inductively coupled plasma mass spectrometry (ICP-MS, Agilent 7,500, Agilent Technology, USA) and high-performance liquid chromatography-inductively coupled plasma mass spectrometry (HPLC-ICP-MS, Agilent 7,500, Agilent Technology, USA), respectively (Yang et al., 2018; Li H. Q. et al., 2025). For quality control, standards and spiked recovery test were included in each batch. The extraction efficiency of the standards was all between 85 and 115%. Besides, the relevant physicochemical properties of porewater, including pH values, electrical conductivity (EC), Fe, Mn and Al concentrations were determined according to the routine methods and our previous studies (Lu, 2000; Yang et al., 2018; Li H. Q. et al., 2025).

### 2.4 Characterization of DOM concentrations and components in porewater

The concentration of DOM in porewater was denoted as that of dissolved organic carbon (DOC), which was measured by a total organic carbon analyzer (Vario TOC, Elementar, Germany) after through a 0.45-μm membrane filter. For the characterization of DOM components, three-dimensional excitation-emission matrix (3DEEM) fluorescence spectra were then characterized using a fluorescence spectrophotometer (F-7000, Hitachi, Japan). Parameter settings were as the following: excitation (Ex) and emission (Em) wavelengths were scanned across ranges of 220–450 nm and 280–550 nm, respectively. Fluorescence intensities were recorded at 5-nm excitation increments and 2-nm emission increments. The scanning speed was set at 12,000 nm min^−1^, and both excitation and emission slit widths were maintained at 5 nm. Raman scattering peaks in the sample EEMs were removed by subtracting the fluorescence signals of ultrapure water. The Rayleigh scattering effect was mitigated by replacing all fluorescence data within two regions (Em ≤ Ex + 30 nm and Em ≥ Ex + 210 nm) with zeros. The fluorescence intensity was calibrated against the average Raman peak of ultrapure water measured at an emission wavelength of 350 nm across an excitation wavelength range of 372–428 nm. Parallel factor analysis (PARAFAC) was employed to decompose the complex DOM mixture into individual fluorescent components, without making any prior assumptions regarding the number of DOM components or their spectral characteristics. All EEM datasets were first compiled into an initial dataset encompassing all samples. The PARAFAC model for DOM components was then constructed and validated using MATLAB (R2019a) with the DOMFluor toolbox, following established protocols. The composition characteristics of DOM were characterized by the proportion of maximum fluorescence intensity (*FI*_max_) of each component, and the biological index (BIX), humification index (HIX) and fluorescence index (*FI*_370_) were also calculated according to the previous studies (An et al., 2026).

### 2.5 Determination of CH_4_ emissions

On the sampling days, both air pumps and three-way stopcocks were turned off for 24 h to collect CH_4_ from the air in the headspace. A gas chromatograph (Agilent 7890A, Agilent Technologies Inc., USA) equipped with a flame ionization detector (FID) was used to quantify CH₄ concentrations in ambient air samples. The emission rate and cumulative emissions of CH_4_ gas were calculated according to previous studies (Xue et al., 2024).

### 2.6 Microbial DNA extraction, quantitative PCR of *mcrA* and *pmoA* genes, as well as 16S rRNA gene amplicon sequencing

Total soil microbial genomic DNA was extracted from 0.5 g of each lyophilized soil sample using the FastDNA SPIN Kit for Soil (MP Biomedicals, USA) following the manufacturer’s protocol. DNA integrity was assessed by electrophoresis on a 0.5% agarose gel stained with GelRed, while DNA concentration and purity (OD₂₆₀/OD₂₈₀ ratio) were determined using a NanoDrop ND-1000 spectrophotometer (Thermo Fisher Scientific, Wilmington, DE, USA). Gene abundances of *mcrA* and *pmoA* were detected using SYBR Green qPCR performed on QuantStudioTM 6 Flex (ABI, USA). Both technical replicates and negative controls were included in each reaction batch. Specific primer sequences, reaction procedures and statistical analyses referred to previous studies (Zheng et al., 2018).

The bacterial and archaeal community structures were characterized. For bacterial community profiling, the V3-V4 hypervariable region of the 16S rRNA gene was amplified by PCR with the universal primer pair 338F/806R (Klindworth et al., 2013). Archaeal communities were targeted by amplifying the V4-V5 region of the archaeal 16S rRNA gene using primers Arch519F/Arch915R (Yang et al., 2016). The resulting PCR amplicons were pooled at equimolar concentrations and subjected to paired-end sequencing (2 × 250 bp) on the Illumina NovaSeq platform. Raw sequencing data were processed using QIIME 2 (Quantitative Insights into Microbial Ecology, version 2), with sequence denoising performed via the DADA2 pipeline (Callahan et al., 2016). Quality-filtered reads were clustered into amplicon sequence variants (ASVs) at 100% sequence similarity against the SILVA reference database. Microbial *α*-diversity across samples was evaluated using the Chao1 richness estimator and Shannon diversity index. For *β*-diversity analysis, non-metric multidimensional scaling (NMDS) ordination based on Bray-Curtis dissimilarities was applied to visualize the microbial community structure differences among different treatments.

### 2.7 Statistical analyses

All datasets were presented as mean values ± standard deviation (SD), with four biological replicates per treatment. Statistical comparisons across different treatments were performed using one-way analysis of variance (ANOVA) followed by Duncan multiple comparisons or the Kruskal-Wallis test via IBM SPSS Statistics 21. *β*-diversity of bacterial and archaeal communities were visualized using NMDS ordination constructed from Bray–Curtis dissimilarity matrices with 999 permutation iterations using the “vegan” package at R platform (version 4.6.1). The PERMANOVA test was performed to evaluate the differences of microbial communities among different treatments by Adonis tool using the “vegan” package (R 4.6.1). Biomarkers were identified through linear discriminant analysis (LDA) effect size (LEfSe), with the Kruskal-Wallis test employed for initial screening (LDA > 3.0, *p* < 0.05) (http://huttenhower.sph.harvard.edu/galaxy). Pearson correlation analysis was performed to evaluate the pairwise linear relationships among CH₄ emissions, soil physicochemical properties, As species, DOM components, CH_4_-metabolic functional genes and biomarkers at genus level. The correlation matrix was visualized as a heatmap using the R package “corrplot” (R 4.6.1). Pairwise Pearson correlation coefficients (r) and corresponding *p* values were calculated, and statistically significant correlations (*p* < 0.05) were annotated in the heatmap. Random forest analysis was carried out by “rfPermute” and “A3” packages in R software (version 4.6.1). All figures and plots were generated using OriginPro 2025b (OriginLab Corporation, Northampton, MA, USA).

### 2.8 Accession numbers of nucleotide sequences

The high-throughput sequencing reads have been submitted to Sequence Read Archive (SRA) database of NCBI with the accession numbers PRJNA1476542 and PRJNA1483373 for bacteria and archaea, respectively.

## 3 Results

### 3.1 Changes of As concentrations and species in porewater under different treatments

Total As concentrations in porewater were determined to evaluate the migration and bioavailability of As in paddy soil under DMAs stress, as well as when biochar was simultaneously applied alongside DMAs. With the extension of incubation time, porewater As concentrations showed similar variation trends across all treatments. Results showed that As concentrations rose sharply within the first 7 days across all treatments (0.26–53.19 mg/L) and then declined gradually to a relatively low level by the end of incubation (0.09–0.87 mg/L) (Figure 1a). As shown in Figure 1a, the addition of DMAs significantly increased the As concentrations in porewater and the change was seriously dose-dependent during the 98-day incubation. On the 3rd day, porewater As concentrations after DMAs addition changed the most compared to the control, which were up to 321.7 and 760.9 times of the control in DMAs60 and DMAs120 treatments, respectively. With the incubation time extended, the changes became weaker. At the end of the incubation, porewater As concentrations in DMAs60 and DMAs120 were 6.2 and 9.9-fold of control, respectively (Figure 1a). Besides, porewater As concentrations in DMAs120 treatments were 2.0–2.5 times of DMAs60 at the early stage of the incubation (0–35 days), which were declined to 1.3–1.7 times at the later stage (Figure 1a). After biochar addition, the concentrations of As in porewater decreased with the increasing application doses of biochar (Figure 1a). At the early incubation stage (0–42 days), porewater As concentrations were 1.0–1.49 times of DMAs120 treatments after 1% biochar applications, while declined to 68.0–76.9% at the later stage. Differently, from the 3rd day on, porewater As concentrations in the 2% biochar treatments were lower than DMAs120 treatments, which significantly declined to 19.9% on the 56th day, and finally stabilized to 45.9% at the end of incubation (Figure 1a).

**Figure 1:**
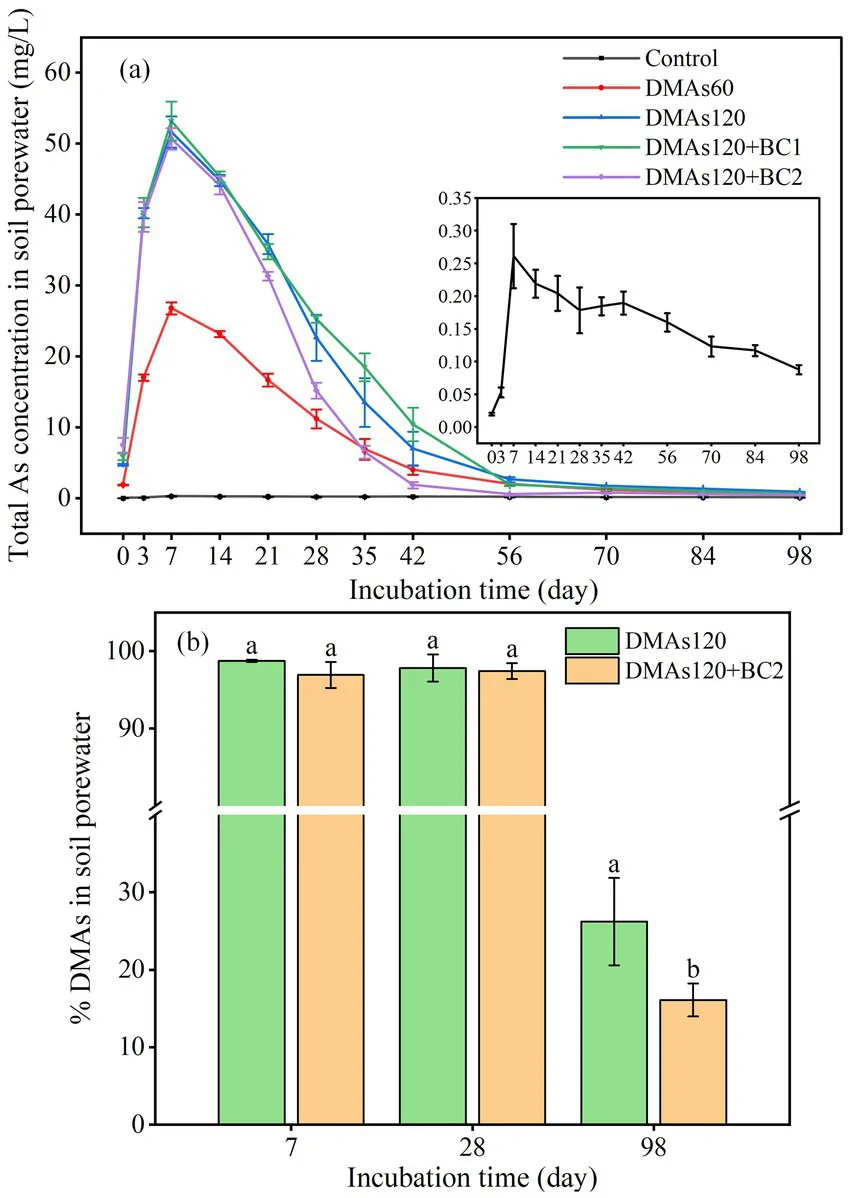
Total arsenic concentrations in porewater under different treatments during the 98-day incubation **(a)** and porewater arsenic species on the 28th and 98th days **(b)**. Different lowercase letters indicate significant differences among different treatments at *p* < 0.05 level.

In addition, As species play a vital role in As mobility and toxicity, thus different As species were also detected in porewater under DMAs120 and DMAs120 + BC2 treatments on days 7, 28 and 98 (Figure 1b). Results showed that the percentages of DMAs (% DMAs) were 98.7 and 98.1% in DMAs120 treatments on days 7 and 28, respectively, while individually declined to 96.9 and 97.4% by 2% biochar additions. At the end of incubation, % DMAs in DMAs treatment decreased to 26.2%, and 2% biochar treatment further significantly decreased it to 16.1% (*p* < 0.05) (Figure 1b). In addition, monomethylarsenate (MMAs) was only detected under DMAs120 treatment on day 28 (Supplementary Table 1). As for inorganic As, the concentrations of arsenate [As(V)] were higher than those of arsenite [As(III)] on days 7 and 28, while on day 98, As(III) were increased to 72.1% and 80.0% under DMAs120 and DMAs120 + BC2 treatments, respectively (Supplementary Table 1).

### 3.2 Variation of CH_4_ emissions under different treatments

The emission rates of CH_4_ were monitored across all treatments during the incubation time, and the cumulative CH_4_ emissions were further calculated to reveal the effects of DMAs on CH_4_ emission from paddy soil, as well as its response to biochar application during the 98-day incubation. As shown in Figure 2a, CH_4_ emission rates of control soil were at a higher level during the first 35 days (maximum of 62.23 μg/(kg·d) on the 35th day). With the extension of incubation time, it declined to 11.79 μg/(kg·d) at the end of incubation. Upon the exposure of DMAs, the CH_4_ emission rates sharply increased during the first 14 days, which were individually up to 176.85 μg/(kg·d) and 213.65 μg/(kg·d) under DMAs60 and DMAs120 treatments. From the 14th day on, the CH_4_ emission rates gradually deceased to 4.07 μg/(kg·d) and 5.88 μg/(kg·d) on the 98th day, respectively. After biochar addition, the variation trends of CH_4_ emission rates were similar to DMAs120 with the incubation time extending, while the absolute emission rates of CH_4_ were individually decreased by 45.34% and 76.94% by 1% and 2% biochar additions on the 14th day.

**Figure 2:**
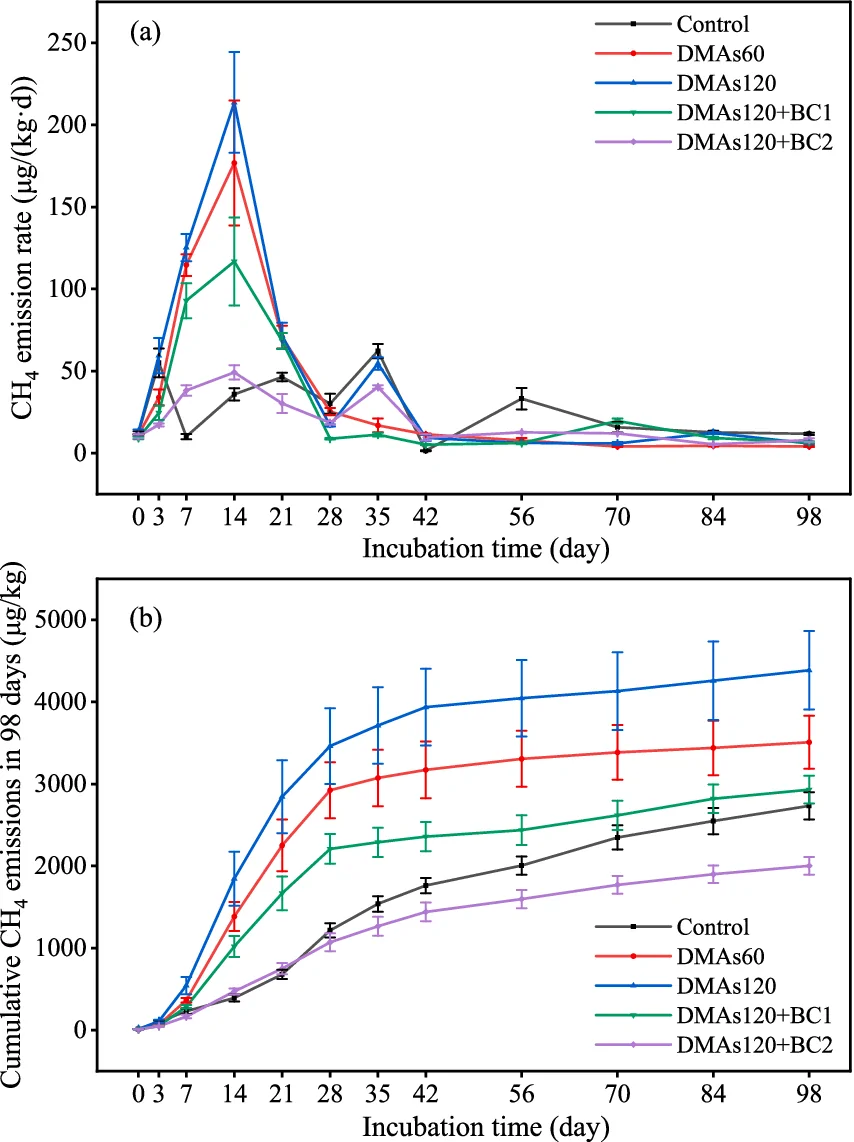
The emission rates **(a)** and cumulative emissions **(b)** of CH_4_ under different treatments during the 98-day incubation.

In addition, the cumulative CH_4_ emissions were significantly dose-dependently increased by DMAs exposure, which individually increased to 1.28 times and 1.60 times of control under DMAs60 and DMAs120 treatments (Figure 2b). However, the addition of biochar significantly decreased the cumulative CH_4_ emissions induced by DMAs exposure, which individually decreased to 66.82% and 45.66% of DMAs120 treatment under 1% and 2% BC treatments (Figure 2b). Besides, after 2% BC addition, the cumulative CH_4_ emission of soil exposed to DMAs120 was decreased by 26.72% compared with the control soil (Figure 2b).

### 3.3 Changes of DOM concentrations and components in porewater

DOM is the key controlling factor for both As biogeochemical cycling and CH_4_ emission in paddy soil. Thus, the DOM concentrations (denoted as DOC concentrations) and components were analyzed across different treatments. Results showed that DOC concentrations continuously increased to a higher level at the early stages (77.2–139.8 mg/L) (0–28 day), while then gradually decreased to a stable level (Figure 3). From the 3rd day on, DMAs exposure increased DOC concentrations in porewater, and this variation was seriously dose-dependent. The most dramatic change induced by DMAs occurred on the 28th day, and the concentrations of DOC were increased to 1.64 and 1.81 times of control in DMAs60 and DMAs120 treatments, respectively (Figure 3). At the end of the incubation, DOC concentrations in DMAs60 and DMAs120 treatments were individually 7.87% and 14.05% higher than that of control (Figure 3). Besides, the DOC concentrations exhibited a decreasing trend induced by biochar application. For example, on the 7th and 21st days, DOC concentrations were reduced 12.06% and 16.58% by 1% biochar addition, respectively (Figure 3). From the 7th day on, DOC concentrations were significantly decreased 12.53–32.07% by 2% biochar (Figure 3).

**Figure 3:**
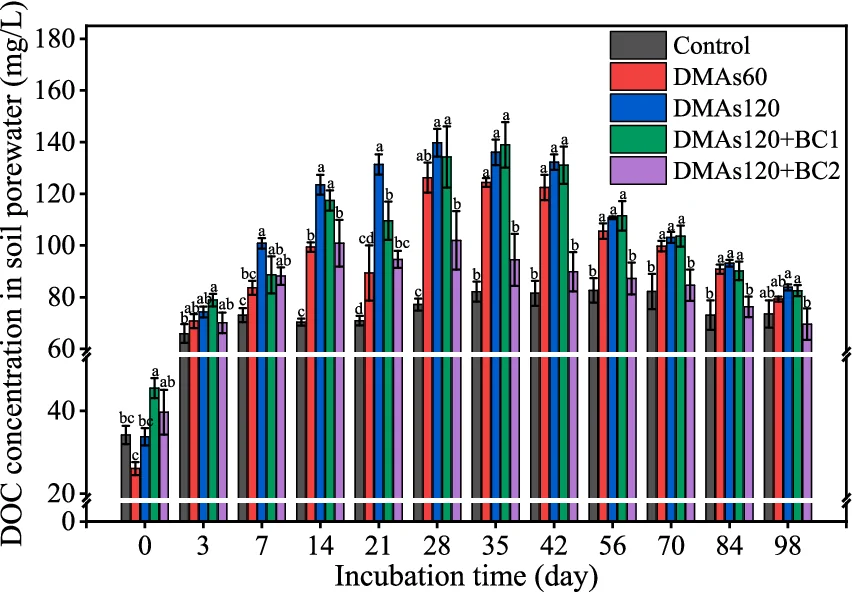
The variations of DOC concentrations in porewater under different treatments during the 98-day incubation. Different lowercase letters indicate significant differences among different treatments at *p* < 0.05 level on the same incubation day.

DOM components were characterized by 3DEEM technology, and four DOM components were identified by PARAFAC analysis, which including Component1 (C1), Component2 (C2), Component 3 (C3) and Component 4 (C4) (Figure 4a; Supplementary Figure 1). In detail, C1 (Ex/Em = 315, 417), C2 (Ex/Em = 280, 338/391), C3 (Ex/Em = 220, 285) and C4 (Ex/Em = 365, 480) were identified as microbial humic-like (UVA humic-like, Peak M), tryptophan-like substances (Peak T), tyrosine-like substances (Peak B) and terrestrial humic-like (visible humic-like, Peak C), respectively (An et al., 2026; Huang et al., 2026). Compared to the control, DMAs120 increased the relative contents of C2 and C3 on the 28th day, while decreased those of C1 and C4 on both 28th and 98th days. After 2% biochar additions, % C1 and % C4 were elevated on both 28th and 98th days, whereas both % C2 and % C3 reduced on the 98th day (Supplementary Table 2; Figure 4b). As shown in Figure 4c, the BIX values ranged from 0.75 to 0.84, and there were no significant changes among different treatments. On both 28th and 98th days, DMAs120 evidently decreased the HIX compared to the control, while 2% biochar significantly increased it. As for *FI*_370_, it ranged from 1.47 to 1.57, while neither DMAs exposure nor biochar addition changed it.

**Figure 4:**
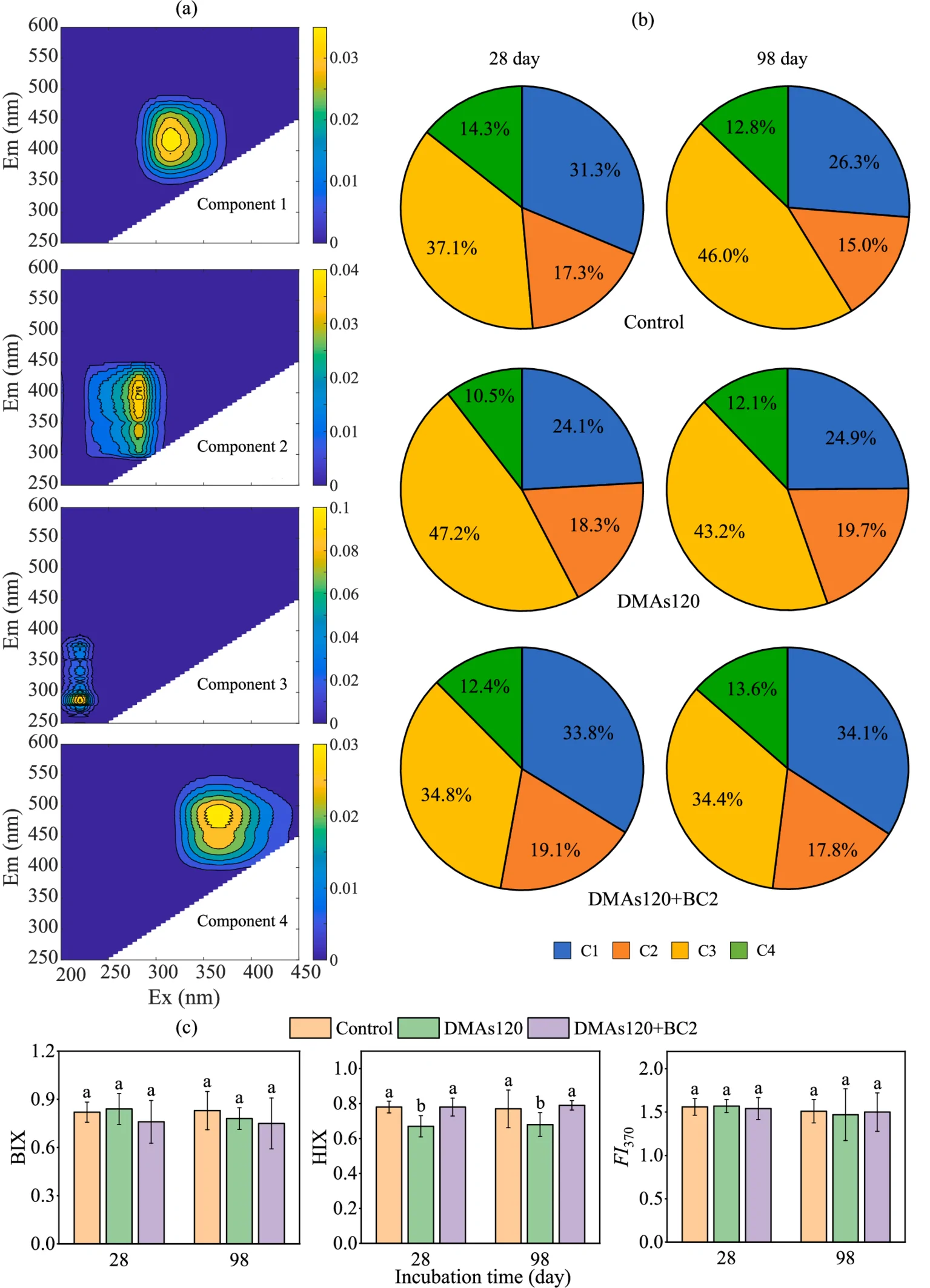
The fluorescent signatures of four fluorescence components output by PARAFAC model **(a)**, the composition of DOM components based on *FI*_max_ **(b)** and the fluorescence indexes of DOM in porewater under different treatments **(c)**. Different lowercase letters indicate significant differences among different treatments at *p* < 0.05 level.

### 3.4 Changes of *mcrA* and *pmoA* gene abundances, and characteristics of microbial community in soil under different treatments

The emissions of CH_4_ depended on both methanogenesis and methanotrophy, which were individually presented as the gene abundances of *mcrA* and *pmoA* in this study. Results showed that DMAs exposure significantly increased the gene copies of *mcrA* and seriously dose-dependent, while biochar dose-dependently reduced the *mcrA* gene abundance, accompanying by the similar level to control after 2% biochar addition (Supplementary Figure 2a). As for the *pmoA* gene, exposure to DMAs exerted no apparent effects on its abundance, whereas biochar markedly elevated the gene’s copy numbers. (Supplementary Figure 2b). Besides, *mcrA*/*pmoA* ratios were further calculated, which indicated that the additions of DMAs dose-dependently increased the *mcrA*/*pmoA* ratios, while biochar evidently decreased this ratio (Supplementary Figure 2c).

Bacteria and archaea are predominant microorganisms that regulate both As cycling process and methane metabolism in paddy soil. Thus, the community compositions of bacteria and archaea in soil were characterized to reveal the response of microorganisms to the exposure of DMAs, as well as DMAs exposure combined with biochar. After quality filtering, 95,736 bacterial ASVs and 947 archaeal ASVs were identified at 100% sequence similarity. *Bacteroidota* (18.92–23.04%), *Chloroflexi* (16.29–22.77%), *Proteobacteria* (10.16–13.38%), *Acidobacteriota* (9.00–13.39%), *Actinobacteriota* (5.08–10.83%) and *Firmicutes* (5.75–10.96%) were dominant bacterial phyla (Supplementary Figure 3). As shown in Figures 5a,b, there was a decrease trend in Chao1 and Shannon indexes due to DMAs exposure, while biochar had no significant effects on the richness and evenness of bacteria by Chao1 and Shannon indexes. The results of NMDS followed by PERMANOVA analysis showed significant changes in bacterial community structures due to both DMAs exposure and biochar additions (R^2^ = 0.3850, Stress = 0.0700) (Figure 5c). LEfSe was performed to pick out bacterial biomarkers under different treatments. A total of 49 bacterial biomarkers were identified from phylum to species level in soil under DMAs120 treatment, which mainly classified into *Actinobacteriota*, *Firmicutes*, *Myxococcota*, *Proteobacteria* and *Bacteroidota* (all LDA > 3.0, *p* < 0.05) (Figure 5d; Supplementary Table 3). As mentioned in 2% biochar treatment, there were 19 bacterial biomarkers which mainly affiliated with *Acidobacteriota*, *Bacteroidota*, *Desulfobacterota* and *Proteobacteria* (all LDA > 3.0, *p* < 0.05) (Figure 5d, Supplementary Table 3).

**Figure 5:**
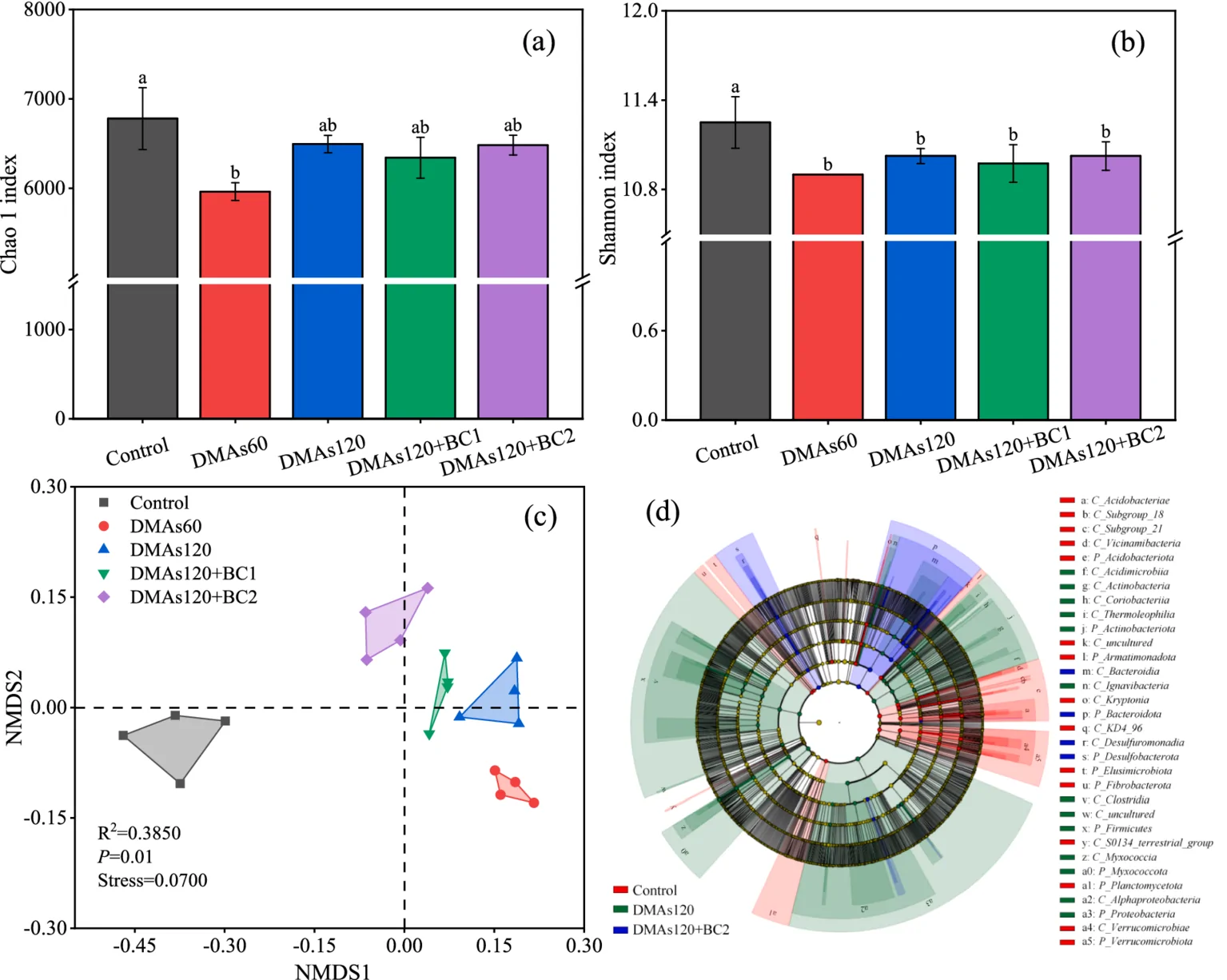
Alpha diversity calculated based on Chao1 **(a)** and Shannon **(b)**, NMDS plots of bacteria based on Bray-Curtis distances under different treatments **(c)**. Linear discriminant analysis (LDA) effect size (LEfSe) (LDA > 3.0, *p* < 0.05) to identify bacterial biomarkers in DMAs120 and DMAs120 + BC2 treatments **(d)**. Different lowercase letters indicate significant differences among different treatments at *p* < 0.05 level. Only biomarkers from phylum to class levels are presented on the LEfSe plot.

For archaea, the predominant phylum was *Crenarchaeota* (98.05–99.24%) (Supplementary Figure 4). Results showed that there were no significant changes in Chao1 index due to DMAs exposure and biochar addition, while Shannon index was significantly increased by DMAs exposure (Figures 6a,b). The results of NMDS showed significant changes in archaeal community structures due to both DMAs exposure and biochar additions (R^2^ = 0.3180, Stress = 0.0348) (Figure 6c). Under DMAs120 and DMAs120 + BC treatments, five and four biomarkers were individually identified from phylum to species level by LEfSe, which were all marked as *Nitrososphaeria* (all LDA > 3.0, *p* < 0.05) (Figure 6d).

**Figure 6:**
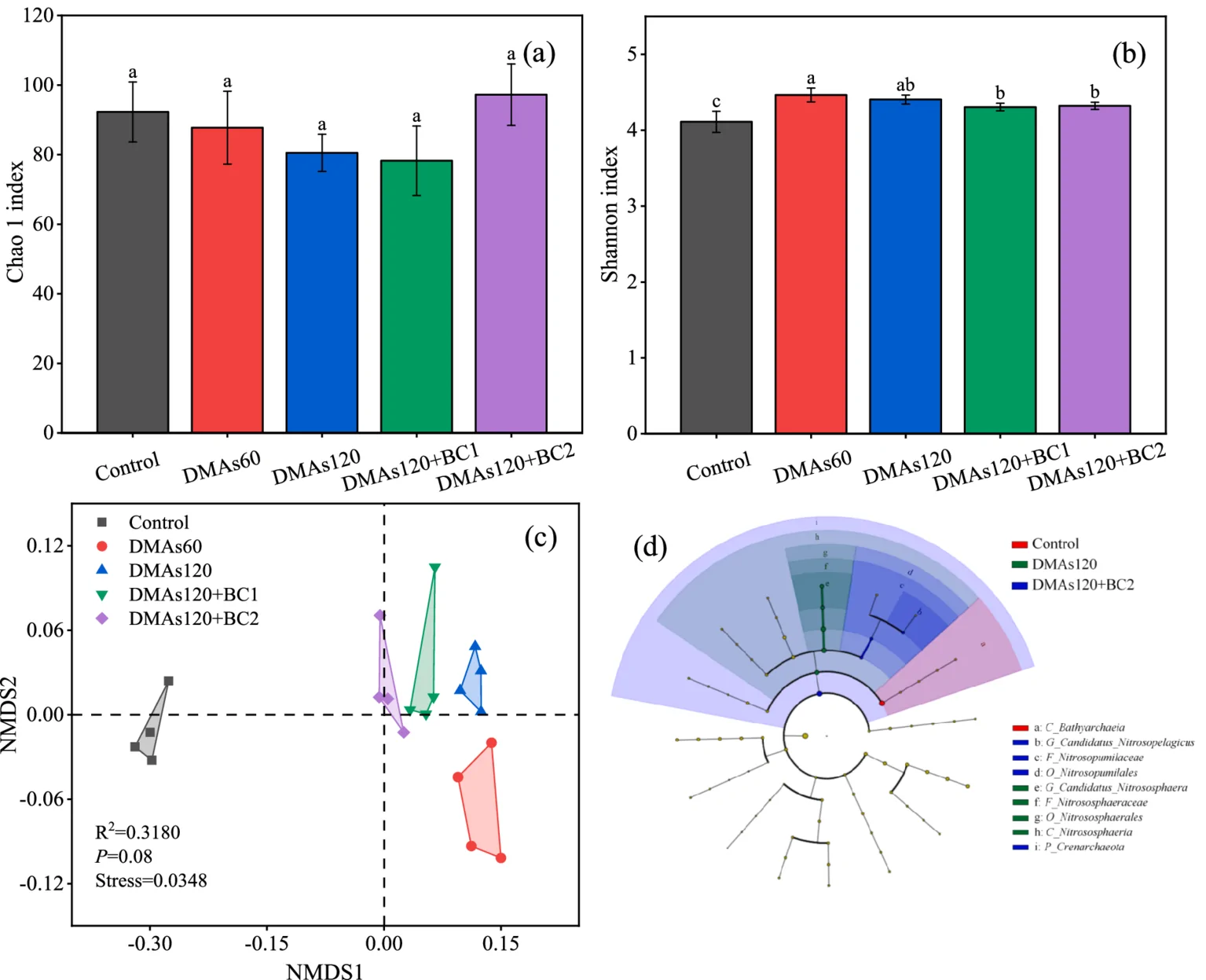
Alpha diversity calculated based on Chao1 **(a)** and Shannon **(b)**, NMDS plots of archaea based on Bray-Curtis distances under different treatments **(c)**. Linear discriminant analysis (*LDA*) effect size (LEfSe) (*LDA* > 3.0, *p* < 0.05) to identify archaeal biomarkers in DMAs120 and DMAs120 + BC2 treatments **(d)**. Different lowercase letters indicate significant differences among different treatments at *p* < 0.05 level. Biomarkers from phylum to genus levels are presented on the LEfSe plot.

### 3.5 Correlation and random forest analyses

Pearson correlation heatmaps were constructed to reveal pairwise linear associations among CH₄ emissions, soil physicochemical properties, As species, DOM fluorescent components, CH_4_-metabolic functional genes and biomarkers at genus level under the DMAs and BC treatments (Figures 7a,b). Under DMAs exposure, CH₄ emissions exhibited significant correlations with multiple soil variables (pH and Fe), As fractions, *mcrA* gene abundance, *mcrA*/*pmoA* ratio, DOM components (C1-C3) and several bacterial genera including *Mycobacterium*, *Flavisolibacter*, *Ignavibacterium*, *Fonticella*, *Gracilibacter*, *Lutispora*, *Anaeromyxobacter* and *Candidatus Nitrososphaera*. Many of these genera showed strong cross-correlations with As species, DOM fractions and CH_4_-related functional genes. Following BC amendment, CH₄ emissions were significantly correlated with soil physicochemical indices (pH, EC and Fe), As(V), DOM components (C1-C3), *mcrA* and *pmoA* gene abundances, *mcrA*/*pmoA* ratio and *Candidatus Nitrosopelagicus*. Notably, the correlation patterns differed markedly between the two treatments, with distinct sets of bacterial genera closely associated with CH₄ emissions in the DMAs and BC groups, implying divergent microbial linkages controlling CH₄ production and oxidation under different amendments.

**Figure 7:**
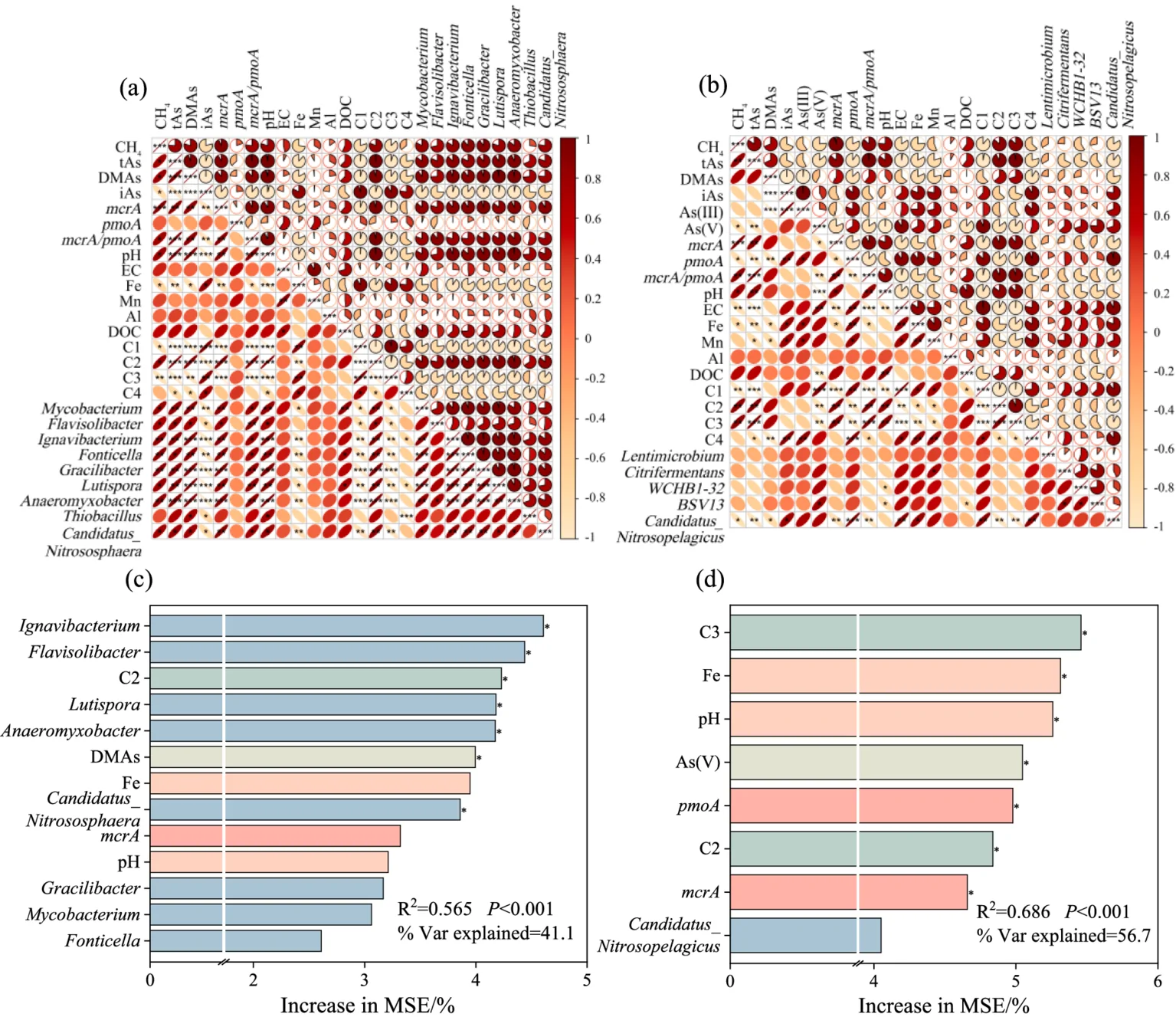
Correlation analyses among CH_4_ emissions, As concentrations (tAs: total arsenic in porewater, iAs: inorganic arsenic in porewater), environmental factors, methane metabolism genes and microbial biomarkers under DMAs120 **(a)** and DMAs120 + BC2 **(b)** treatments, as well as random forest analyses revealing the variation of CH_4_ under DMAs120 **(c)** and DMAs120 + BC2 **(d)** treatments (*p < 0.05).

Furthermore, random forest analysis was applied to quantify the relative importance of explanatory variables regulating CH₄ emissions under the DMAs and BC treatments, respectively. For the DMAs treatment model, the predictor set explained 41.1% of the total variance in CH₄ emissions (R^2^ = 0.565, *p* < 0.001). Significant explanatory variables included *Ignavibacterium*, *Flavisolibacter*, C2, *Lutispora*, *Anaeromyxobacter*, DMAs, and *Candidatus Nitrososphaera* (*p* < 0.05). Among them, *Ignavibacterium* exhibited the highest importance (%IncMSE = 4.61, *p* = 0.034), followed by *Flavisolibacter* (%IncMSE = 4.44, *p* = 0.038) and C2 (%IncMSE = 4.23, *p* = 0.037). Variables including Fe, *mcrA*, pH, *Gracilibacter*, *Mycobacterium* and *Fonticella* showed non-significant contributions (*p* > 0.05) (Figure 7c). For the BC amendment model, predictors accounted for 56.7% of the variance in CH₄ emissions (R^2^ = 0.686, *p* < 0.001). The dominant significant predictors were C3, Fe, pH, As(V), *pmoA*, C2, and *mcrA* (*p* < 0.05). C3 contributed the greatest importance (%IncMSE = 5.46, *p* = 0.024), followed by Fe (%IncMSE = 5.31, *p* = 0.012). In contrast, *Candidatus Nitrosopelagicus* exerted non-significant effects on CH₄ emissions (*p* > 0.05) (Figure 7d).

## 4 Discussion

Our results revealed that DMAs exposure stimulated CH₄ emissions from paddy soil, while biochar amendments effectively mitigated such stimulatory impact. Random forest analyses revealed that the key drivers of CH₄ emissions shifted from sensitive microbial genera and DOM fractions under DMAs exposure to DOM components, soil physicochemical properties, As speciation, and CH_4_-metabolic functional genes following biochar addition (Figures 7c,d).

### 4.1 The exposure of DMAs significantly stimulated the emissions of CH_4_ from paddy soil mainly through DOM-microbe interactions

DMAs addition dose-dependently elevated total As concentrations in porewater, where DMAs dominated the As speciation at early incubation stage and was gradually transformed over time, resulting in a substantial increase in the proportion of inorganic As(III) in the late incubation period (Figure 1; Supplementary Table 1). Simultaneously, the cumulative emissions of CH_4_ were also evidently increased by DMAs input during the 98-day incubation (Figure 2b). We also found that exogenous DMAs stress triggered a serious of changes in multiple environmental and microbial indicators (Figures 3–6; Supplementary material). In brief, throughout incubation, DMAs treatment increased porewater pH possibly via proton consumption during DMAs demethylation and microbial Fe(III) reduction (Freitas et al., 2022). Dissolved Fe accumulated early but declined at the end probably owing to Fe-As co-precipitation and pH-promoted Fe immobilization on solid minerals (Voegelin et al., 2019). DMAs also raised porewater DOC (peaking on day 28), increased labile tryptophan-like C2 and tyrosine-like C3, as well as lowered HIX. Anaerobic fermentative and dissimilatory iron-reducing bacteria, as well as *Candidatus Nitrososphaera* were also enriched. Moreover, DMAs elevated *mcrA* gene abundance but barely changed *pmoA*, thus increasing the *mcrA*/*pmoA* ratio. These combined changes caused dose-dependent increases in cumulative CH₄ emissions.

To clarify the core mechanisms driving CH₄ emissions, random forest analysis was performed for the DMAs treatment. The model explained 41.1% of the total variance in CH₄ emissions (R^2^ = 0.565, *p* < 0.001) (Figure 7c). *Ignavibacterium*, *Flavisolibacter*, C2, *Lutispora*, *Anaeromyxobacter*, DMAs and *Candidatus Nitrososphaera* were identified as significant predictors, demonstrating that DOM-microbe interactions constituted the core pathway regulating CH_4_ emissions under DMAs stress. The key bacterial taxa with predictive importance could be grouped into two functional guilds. The first group represented anaerobic fermentative bacteria (*Flavisolibacter*, *Lutispora*), which degrade polysaccharides and protein-derived DOM to produce methanogenic precursors including acetate and hydrogen (Wegner and Liesack, 2016; Liu et al., 2022; Cheng et al., 2024; Luo et al., 2025). The second group comprised dissimilatory iron-reducers coupled with metabolism of soil organic matter (SOM) (*Ignavibacterium*, *Anaeromyxobacter*). These taxa could utilize labile DOM as electron donors for Fe(III) reduction and simultaneously decompose organic substrates to supply precursors for methanogenesis (Bei et al., 2021). DMAs toxicity might induce microbial cell lysis and SOM desorption, raising the relative abundance of tryptophan-like labile DOM component C2. As bioavailable carbon and electron donors, elevated C2 selectively enriched the above-mentioned functional bacterial guilds. These microorganisms continuously supplied methanogenic substrates via SOM mineralization and indirectly drove methanogenesis. As a significant predictor, DMAs itself reflected toxic stress effects that disturbed SOM turnover and microbial community assembly, and its degradation was dominated by the production of As(III), which imposed toxic pressure on microorganisms and modified DOM-As complexation to regulate DOM bioavailability. The archaeal biomarker *Candidatus Nitrososphaera* indirectly modulated CH₄ metabolism by mediating ammonia oxidation and altering microhabitat conditions. In summary, there existed bidirectional feedback between DOM and microorganisms, where shifts in DOM properties drove the enrichment of functional bacteria that further degraded and transformed DOM and reshaped porewater DOM composition.

Additionally, several variables exhibited pronounced treatment-induced changes and showed significant correlations with CH₄ emissions according to Pearson correlation analyses, yet they were not selected as independent significant predictors in the random forest model, especially including *mcrA*, pH and Fe (Figure 7c). It indicated that these variables mainly acted as contextual environmental regulators rather than primary drivers. Although the gene abundance of *mcrA* and the *mcrA*/*pmoA* ratio increased substantially under DMAs exposure, *mcrA* showed no independent predictive power. It suggested that DMAs did not directly target methanogenic archaea; enhanced CH₄ production was largely attributed to elevated substrate supply originating from upstream fermentative and iron-reducing bacteria, instead of direct stimulation by *mcrA* abundance. The DMAs-induced pH elevation modified the dissociation state of DOM molecules, improved the bioavailability of certain DOM fractions, and optimized enzyme activities of anaerobic microorganisms, thereby ameliorating microhabitat conditions for functional microbes (Li et al., 2018; Man and Simpson, 2025). Besides, Fe exhibited distinct temporal dynamics. Specifically, the proliferation of iron-reducing bacteria at the early stage promoted the reduction of Fe(III) to dissolved Fe(II), which competed for electron donors with methanogenesis and inhibited CH_4_ production. At the late incubation stage, dissolved porewater Fe decreased owing to co-precipitation, thereby alleviating this electron competition and indirectly facilitating methanogenesis (Li H. Y. et al., 2025). In addition, As speciation in porewater dynamically shifted over incubation time, dominated by DMAs at early stages and by As(III) afterwards. Different As species imposed toxic stress on microorganisms and altered DOM bioavailability via As-DOM complexation, jointly participating in the regulatory network. Collectively, pH, Fe, As speciation and *mcrA* served as background environmental factors that amplified or modulated DOM-microbe interactions.

In summary, DMAs enhanced CH_4_ emissions not by directly stimulating methanogenic archaea. DMAs input first increased As bioavailability in porewater and triggered SOM desorption and cell lysis, resulting in elevated DOC and accumulation of labile DOM component C2. Meanwhile, DMAs demethylation and iron-reduction reactions consumed H^+^ and raised porewater pH. Dissolved Fe(II) was released in early phases, whereas Fe(II)-As co-precipitation decreased porewater Fe concentrations at the late stage. Labile DOM further selected and enriched fermentative and dissimilatory iron-reducing bacteria, which in turn modified DOM properties, forming bidirectional coupled interactions that regulated methanogenesis through substrate supply as well as synergistic and competitive inter-species relationships. pH, Fe redox-precipitation processes and As-speciation transformation were embedded within this pathway and jointly mediated elevated CH₄ emissions under DMAs stress.

### 4.2 Regulation of biochar on CH_4_ mitigation under DMAs stress in paddy soils

Biochar pyrolyzed at 450 °C was selected as the soil amendment in this study. The 450 °C pyrolysis temperature falls within the medium-low thermal regime. Biochar produced under this condition retains relatively abundant oxygen-containing functional groups, providing abundant adsorption sites for complexing heavy metals. Meanwhile, it preserves a certain proportion of labile organic fractions, avoiding the drawbacks of high-temperature biochar, such as excessive aromatization, low labile organic carbon content, and strong suppression of microbial activity. Accordingly, 450 °C represents a widely adopted pyrolysis temperature for paddy soil remediation (Nan et al., 2021).

Our results showed that biochar applied at a rate of 2% significantly reduced total As concentrations in porewater and lowered the proportion of DMAs in dissolved As, while increasing the concentrations of inorganic As(III) and As(V). This observation implied that biochar likely promoted the demethylation of DMAs, converting methylated As into inorganic As species, which could be verified in future studies. Biochar amendment increased the relative abundance of humic-like DOM (C1, C4) and decreased labile biogenic DOM (C2, C3). Although raw biochar itself is highly alkaline, porewater pH had already been elevated under DMAs treatment relative to control. Therefore, biochar amendment did not substantially increase pH and even led to a slight pH decrease in the late incubation period. Biochar also markedly increased the abundance of *pmoA* and reduced *mcrA* and the *mcrA*/*pmoA* ratio, ultimately mitigating DMAs-induced cumulative CH₄ emissions to a level even below the control treatment. The porous structure of biochar offered suitable microhabitats and selectively enriched five core genera with distinct biogeochemical functions (*Lentimicrobium*, *Citrifermentans*, *WCHB1-32*, *BSV13* and *Candidatus Nitrosopelagicus*).

Random forest analysis revealed that the key predictors governing CH₄ emissions shifted to C3, Fe, pH, As(V), *pmoA*, C2 and *mcrA* under biochar amendment. Microorganisms are the direct mediators of CH_4_ metabolism, and the directional regulation of microbial communities by biochar represents the core mechanism underlying its effect on CH₄ emissions. The appearance of *mcrA* and *pmoA* in the key predictor set indicated bidirectional regulation by biochar, which simultaneously suppressed methanogenesis (*mcrA*) and stimulated methane oxidation (*pmoA*), thereby reducing net soil CH₄ emissions. Besides, C2, C3, Fe, pH and As(V) collectively regulated net CH₄ emissions through divergent pathways. Firstly, the labile biogenic DOM fractions C2 and C3 primarily governed substrate availability for methanogenesis. The reduced concentrations of these compounds limited carbon supply for fermentative microorganisms and subsequently constrained the production of acetate and hydrogen, which are the key substrates for methanogens (Brüggenwirth et al., 2024). Secondly, as a dissimilatory iron-reducing bacterium, the metabolic activity of *Citrifermentans* drove the dissolution and reduction of solid-phase Fe(III), contributing to elevated porewater Fe concentrations (List et al., 2019). Fe exerted bidirectional controls over CH₄ cycling, in which Fe(III) suppressed methanogenesis via electron competition and supported CH_4_ consumption by acting as a terminal electron acceptor for iron-dependent anaerobic oxidation of methane (AOM) (Fan et al., 2020). The enriched genus *BSV13*, associated with Fe redox metabolism, further participated in Fe transformation and strengthened this electron competition effect (Dong et al., 2023). Thirdly, As(V) mainly facilitated CH_4_ oxidation by serving as a terminal electron acceptor for arsenic-coupled AOM, while indirectly modulating methanogenic communities through alterations in As toxicity and mineral immobilization (Shi et al., 2020; Jiang et al., 2024). Besides, pH functioned as a master environmental regulator, modifying microbial living conditions, DOM dissociation characteristics, and the speciation of Fe minerals and As, thereby indirectly affecting both methanogenesis and methane oxidation (Liao et al., 2020). Meanwhile, *Candidatus Nitrosopelagicus* (an ammonia-oxidizing archaeon) mediated nitrification, altered soil nitrogen availability and local pH, and indirectly modulated As transformation and the production of AOM electron acceptors (Li R. F. et al., 2025). Together with the functional genes *mcrA* and *pmoA*, these physicochemical and DOM-related variables formed a coupled network governing CH₄ dynamics under biochar amendment.

Overall, biochar effectively reversed the promotional effect of DMAs on soil CH₄ emissions via multi-pathway synergistic regulation. On the one hand, biochar accelerated DMAs demethylation to increase inorganic As(V) levels, which served as critical electron acceptors for AOM and facilitated CH_4_ consumption. On the other hand, biochar restructured DOM components by depleting labile C2 and C3 fractions to reduce methanogenic substrate supply, while selectively enriching functional microbial communities including fermentative, iron-reducing and ammonia-oxidizing taxa. Specifically, the enriched *Citrifermentans* promoted Fe(III) reduction to elevate porewater Fe concentrations, which intensified electron competition against methanogenesis and supported Fe-dependent AOM, whereas *Candidatus Nitrosopelagicus*-mediated nitrification modulated microenvironmental pH and As speciation to further optimize the CH_4_ cycling environment. Coupled with the downregulated *mcrA* abundance and upregulated *pmoA* abundance, the integrated coupling of microbial community succession, DOM transformation, Fe/As speciation evolution and physicochemical changes thoroughly reshaped the dominant regulatory mechanism of CH₄ emissions. This study demonstrated that biochar could act as an efficient soil amendment to mitigate methylated arsenic-triggered CH_4_ overproduction in flooded paddy soils.

This study has several limitations. The incubation experiment was carried out using only a single paddy soil under laboratory anaerobic conditions. Paddy soils differ widely in native Fe concentration, background DOM pools, indigenous microbial communities and baseline pH values. In addition, field conditions involve complex environmental processes including rice root oxygen release, root exudation and drying-rewetting cycles caused by rainfall. Therefore, the mechanistic conclusions obtained herein require further verification through multi-soil incubations and in-situ field trials, and the practical performance of biochar application should also be evaluated under field conditions. Furthermore, random forest serves only as an exploratory and predictive tool and cannot replace structural equation modeling (SEM) for causal pathway analysis. The limited sample size in this study prevents reliable quantitative causal inference via SEM. Although random forest was used to assess the relative importance of driving factors and predict microbial community responses, it cannot separate direct from indirect variable effects, unlike SEM. We recognize that the lack of SEM-based causal validation represents a key limitation of our study. Future work will increase sample size to develop a well-fitted SEM model and systematically clarify how environmental variables and microbial communities drive shifts in CH_4_ emissions.

## 5 Conclusion

This study revealed that DMAs stimulated CH₄ emissions from paddy soils mainly by increasing labile C2 and C3 DOM, enriching anaerobic fermentative microbes, and improving substrate availability for methanogenesis, which were also corresponding with the increase of *mcrA* gene. In contrast, biochar amendment effectively reversed DMAs-induced CH₄ overproduction. Biochar promoted DMAs demethylation to produce more As(V), which could act as a terminal electron acceptor for anaerobic CH₄ oxidation. It also reduced labile DOM substrates and selectively enriched key functional microbes. Specifically, iron-reducing *Citrifermentans* increased porewater Fe levels to strengthen electron competition and Fe-dependent CH₄ oxidation, while ammonia-oxidizing *Candidatus Nitrosopelagicus* indirectly modulated CH₄ cycling by altering soil pH, As speciation and nitrification. Combined with decreased *mcrA* and elevated *pmoA* abundances, biochar restructured the CH₄ regulatory system from DOM-microbe interaction to integrated physicochemical, As, DOM and genetic regulation. This work reveals the As-C coupling in methylated As-contaminated paddy soils and verifies the capacity of medium-temperature biochar for concurrent As remediation and greenhouse gas mitigation. Further multi-soil and field investigations are needed to validate the generalizability of these laboratory-based findings.

## Data Availability

The datasets presented in this study can be found in online repositories. The names of the repository/repositories and accession number(s) can be found in the article/Supplementary material.
