# Supplementary material for: Biochar mitigates dimethylarsenate-stimulated methane emissions mediated by DOM-microbe interactions in paddy soils
Source: Front Microbiol. 2026 Sep 18;17:1958612. doi: 10.3389/fmicb.2026.1958612 (PMC13630724; doi:10.3389/fmicb.2026.1958612)
Supplement: Supplementary file 1 [file Data_Sheet_1.docx]

Supplementary Material

# 1 Supplementary Data

**Changes of physicochemical factors in porewater under different treatments**

Soil porewater pH, EC, Fe, Mn. And Al concentrations were also determined.

It can be seen from the results that the pH value of soil porewater in all treatments showed an increasing trend at the early stage of incubation and reached the maximum value (7.83-8.20) on the 28th day of incubation, and then decreased to a stable level at the end of incubation, the pH value of DMAs60 and DMAs120 treatments were significantly higher than that of the control, but there was no difference between the two groups. However, DMAs120+BC1 and DMAs120+BC2 treatments were significantly improved compared with the DMAs120 treatment (Supplementary Table 4).

The EC values further confirmed the improvement effect of biochar, which may be related to the rich mineral content of biochar and its enhancement of soil nutrient retention capacity. The trend of EC porewater value was similar to that of pH value, reaching the maximum value (2.48-3.51 mS/cm) on the 7th day of incubation, the EC value of DMAs120+BC1 treatment was the highest during the incubation, which was significantly higher than that of other treatments (Supplementary Table 5).

The dynamic change of soil porewater Fe concentration showed that there was no significant difference among the treatments at the beginning of incubation, and the maximum value (20.29-53.85 mg/L) was reached on the 3rd day in all treatments except DMAs120+BC2 treatment. At the end of incubation, DMAs significantly decreased Fe concentrations in porewater, while biochar evidently increased the porewater Fe concentrations (Supplementary Table 6).

The results of Mn concentrations in soil porewater showed that the first concentration peak appeared on the 3rd day in all treatments, and the highest concentration appeared in the treatment of DMAs120+BC1 (16.02 ± 4.10 mg/L) The effect of DMAs120+BC2 on the dissolution of Mn was the most prominent and stable, and the concentration reached 6.75 times (12.29 ± 1.2 mg/L) of the control group on the 7th day, from 70 to 98 days, DMAs120 + BC1 was significantly higher than that of DMAs120 treatment (Supplementary Table 7).

The difference of Al concentrations among treatments was relatively small, and the highest value appeared between 56 and 84 days (531.64-599.98 μg/L), which was maintained at 499.40-508.89 μg/L at the end of incubation (Supplementary Table 8).

# 2 Supplementary Figures and Tables

## 2.1 Supplementary Figures

**Supplementary Figure 1.** The split-half validation results of the four DOM components (excitation (left) and emission (right) loading spectra.

**Supplementary Figure 2.** Gene abundances of *mcrA* (a) and *pmoA* (b) in soil, as well as *mcrA*/*pmoA* (c) ratio under different treatments.

**Supplementary Figure 3.** Changes in bacterial phylum (a) and genus (b) levels under different treatments.

**Supplementary Figure 4.** Changes in archaeal phylum (a), class (b) and genus (c) levels under different treatments.

## 2.2 Supplementary Tables

**Supplementary Table 1.** Proportion of different arsenic species in soil porewater (%)

| Arsenic species | Day 7 | | Day 28 | | Day 98 | |
| --- | --- | --- | --- | --- | --- | --- |
|  | DMAs120 | DMAs120+BC2 | DMAs120 | DMAs120+BC2 | DMAs120 | DMAs120+BC2 |
| As(Ⅲ) | 0.4±0.2a | 0.1±0.1b | 0.2±0.0b | 0.6±0.1a | 72.1±3.6a | 80.0±9.2a |
| As(Ⅴ) | 0.9±0.2a | 3.0±1.6a | 1.3±0.4a | 1.9±0.5a | 1.7±0.36b | 3.9±0.8a |
| MMAs | Undetected | Undetected | 0.4±0.0 | Undetected | Undetected | Undetected |

Note: Different lowercase letters indicated significant difference among different treatments at the same incubation stages (*P* < 0.05). Data are means ± SD, n = 4.

**Supplementary Table 2.** The maximum fluorescence intensities (*FI*_max_) of DOM components in soil porewater

|  | Control | | | | DMAs120 | | | | DMAs120+BC2 | | | |
| --- | --- | --- | --- | --- | --- | --- | --- | --- | --- | --- | --- | --- |
|  | Component1 | Component2 | Component3 | Component4 | Component1 | Component2 | Component3 | Component4 | Component1 | Component2 | Component3 | Component4 |
| Day  28 | 15.9 | 8.8 | 18.8 | 7.3 | 20.6 | 15.6 | 40.4 | 8.9 | 13.9 | 7.9 | 14.3 | 5.1 |
| Day  98 | 13.1 | 7.5 | 22.9 | 6.4 | 15.7 | 12.5 | 27.3 | 7.7 | 13.6 | 7.1 | 13.7 | 5.4 |

**Supplementary Table 3.** Bacterial biomarkers identified by LEfSe analysis under different treatments

| Biomarker names | Phylum | Group | LDA values | *P* values |
| --- | --- | --- | --- | --- |
| *F_67_14* | *Actinobacteriota* | DMAs120 | 3.70 | 0.0154 |
| *O_Solirubrobacterales* | *Actinobacteriota* | DMAs120 | 3.76 | 0.0097 |
| *O_Corynebacteriales* | *Actinobacteriota* | DMAs120 | 3.15 | 0.0124 |
| *F_Solirubrobacteraceae* | *Actinobacteriota* | DMAs120 | 3.03 | 0.0097 |
| *C_Acidimicrobiia* | *Actinobacteriota* | DMAs120 | 3.21 | 0.0387 |
| *F_Intrasporangiaceae* | *Actinobacteriota* | DMAs120 | 3.46 | 0.0183 |
| *O_Micrococcales* | *Actinobacteriota* | DMAs120 | 3.53 | 0.0209 |
| *C_Coriobacteriia* | *Actinobacteriota* | DMAs120 | 3.65 | 0.0072 |
| *C_Actinobacteria* | *Actinobacteriota* | DMAs120 | 3.90 | 0.0154 |
| *O_OPB41* | *Actinobacteriota* | DMAs120 | 3.67 | 0.0072 |
| *C_Thermoleophilia* | *Actinobacteriota* | DMAs120 | 3.92 | 0.0072 |
| *P_Actinobacteriota* | *Actinobacteriota* | DMAs120 | 4.32 | 0.0072 |
| *G_Mycobacterium* | *Actinobacteriota* | DMAs120 | 3.05 | 0.0230 |
| *O_Gaiellales* | *Actinobacteriota* | DMAs120 | 3.30 | 0.0154 |
| *O_Ignavibacteriales* | *Bacteroidota* | DMAs120 | 3.76 | 0.0307 |
| *S_uncultured_bacterium* | *Bacteroidota* | DMAs120 | 3.58 | 0.0209 |
| *G_Flavisolibacter* | *Bacteroidota* | DMAs120 | 3.61 | 0.0244 |
| *F_Ignavibacteriaceae* | *Bacteroidota* | DMAs120 | 3.56 | 0.0183 |
| *G_Ignavibacterium* | *Bacteroidota* | DMAs120 | 3.59 | 0.0183 |
| *O_Chitinophagales* | *Bacteroidota* | DMAs120 | 3.86 | 0.0387 |
| *F_Chitinophagaceae* | *Bacteroidota* | DMAs120 | 3.74 | 0.0387 |
| *C_Ignavibacteria* | *Bacteroidota* | DMAs120 | 3.57 | 0.0307 |
| *G_Fonticella* | *Firmicutes* | DMAs120 | 3.37 | 0.0172 |
| *O_Peptostreptococcales_Tissierellales* | *Firmicutes* | DMAs120 | 3.16 | 0.0183 |
| *F_Hungateiclostridiaceae* | *Firmicutes* | DMAs120 | 3.03 | 0.0497 |
| Biomarker names | Phylum | Group | LDA values | *P* values |
| *C_Clostridia* | *Firmicutes* | DMAs120 | 4.15 | 0.0172 |
| *F_Caloramatoraceae* | *Firmicutes* | DMAs120 | 3.45 | 0.0172 |
| *O_Clostridiales* | *Firmicutes* | DMAs120 | 3.75 | 0.0209 |
| *P_Firmicutes* | *Firmicutes* | DMAs120 | 4.31 | 0.0124 |
| *F_Gracilibacteraceae* | *Firmicutes* | DMAs120 | 3.52 | 0.0072 |
| *C_uncultured* | *Firmicutes* | DMAs120 | 3.30 | 0.0183 |
| *G_Gracilibacter* | *Firmicutes* | DMAs120 | 3.27 | 0.0097 |
| *G_Lutispora* | *Firmicutes* | DMAs120 | 3.05 | 0.0132 |
| *F_Clostridiaceae* | *Firmicutes* | DMAs120 | 3.35 | 0.0373 |
| *S_uncultured_bacterium* | *Firmicutes* | DMAs120 | 3.36 | 0.0172 |
| *G_Anaeromyxobacter* | *Myxococcota* | DMAs120 | 3.85 | 0.0072 |
| *C_Myxococcia* | *Myxococcota* | DMAs120 | 3.90 | 0.0072 |
| *P_Myxococcota* | *Myxococcota* | DMAs120 | 3.86 | 0.0172 |
| *S_uncultured_bacterium* | *Myxococcota* | DMAs120 | 3.09 | 0.0124 |
| *F_Anaeromyxobacteraceae* | *Myxococcota* | DMAs120 | 3.81 | 0.0072 |
| *O_Myxococcales* | *Myxococcota* | DMAs120 | 3.86 | 0.0072 |
| *O_Rhizobiales* | *Proteobacteria* | DMAs120 | 3.43 | 0.0230 |
| *P_Proteobacteria* | *Proteobacteria* | DMAs120 | 4.25 | 0.0366 |
| *C_Alphaproteobacteria* | *Proteobacteria* | DMAs120 | 3.99 | 0.0154 |
| *F_Hydrogenophilaceae* | *Proteobacteria* | DMAs120 | 3.42 | 0.0263 |
| *G_Thiobacillus* | *Proteobacteria* | DMAs120 | 3.43 | 0.0263 |
| *O_Defluviicoccales* | *Proteobacteria* | DMAs120 | 3.03 | 0.0124 |
| *F_Defluviicoccaceae* | *Proteobacteria* | DMAs120 | 3.00 | 0.0154 |
| *F_Comamonadaceae* | *Proteobacteria* | DMAs120 | 3.47 | 0.0230 |
| *O_AKIW659* | *Acidobacteriota* | DMAs120+BC2 | 3.20 | 0.0230 |
| *O_Bacteroidales* | *Bacteroidota* | DMAs120+BC2 | 4.38 | 0.0249 |
| Biomarker names | Phylum | Group | LDA values | *P* values |
| *P_Bacteroidota* | *Bacteroidota* | DMAs120+BC2 | 4.40 | 0.0230 |
| *F_Prolixibacteraceae* | *Bacteroidota* | DMAs120+BC2 | 3.87 | 0.0124 |
| *S_uncultured_bacterium* | *Bacteroidota* | DMAs120+BC2 | 3.34 | 0.0072 |
| *S_bacterium_enrichment_culture_clone_GD_A_23* | *Bacteroidota* | DMAs120+BC2 | 3.70 | 0.0244 |
| *F_Bacteroidetes_vadinHA17* | *Bacteroidota* | DMAs120+BC2 | 4.31 | 0.0263 |
| *G_BSV13* | *Bacteroidota* | DMAs120+BC2 | 3.28 | 0.0154 |
| *C_Bacteroidia* | *Bacteroidota* | DMAs120+BC2 | 4.40 | 0.0124 |
| *G_Lentimicrobium* | *Bacteroidota* | DMAs120+BC2 | 3.77 | 0.0230 |
| *G_WCHB1_32* | *Bacteroidota* | DMAs120+BC2 | 3.74 | 0.0154 |
| *P_Desulfobacterota* | *Desulfobacterota* | DMAs120+BC2 | 3.74 | 0.0230 |
| *G_Citrifermentans* | *Desulfobacterota* | DMAs120+BC2 | 3.56 | 0.0124 |
| *F_Geobacteraceae* | *Desulfobacterota* | DMAs120+BC2 | 3.64 | 0.0244 |
| *C_Desulfuromonadia* | *Desulfobacterota* | DMAs120+BC2 | 3.67 | 0.0244 |
| *O_Geobacterales* | *Desulfobacterota* | DMAs120+BC2 | 3.66 | 0.0244 |
| *F_Magnetospirillaceae* | *Proteobacteria* | DMAs120+BC2 | 3.19 | 0.0230 |
| *F_Gallionellaceae* | *Proteobacteria* | DMAs120+BC2 | 3.54 | 0.0154 |
| *O_Rhodospirillales* | *Proteobacteria* | DMAs120+BC2 | 3.20 | 0.0230 |

**Supplementary Table 4.** pH of soil porewater

| Treatments  Incubation time | Control | DMAs60 | DMAs120 | DMAs120+BC1 | DMAs120+BC2 |
| --- | --- | --- | --- | --- | --- |
| Day 0 | 7.52±0.08c | 7.54±0.08c | 7.57±0.02c | 7.71±0.06b | 7.88±0.06a |
| Day 3 | 7.42±0.10c | 7.57±0.10b | 7.55±0.06b | 7.75±0.06a | 7.75±0.06a |
| Day 7 | 7.48±0.12b | 7.60±0.20ab | 7.58±0.08ab | 7.65±0.08ab | 7.74±0.14a |
| Day 14 | 7.49±0.04b | 7.62±0.08ab | 7.72±0.08a | 7.73±0.10a | 7.77±0.20a |
| Day 21 | 7.50±0.18b | 7.77±0.22a | 7.86±0.16a | 7.78±0.08a | 7.75±0.12a |
| Day 28 | 7.83±0.02b | 8.09±0.08a | 8.20±0.20a | 8.17±0.10a | 8.18±0.10a |
| Day 35 | 7.20±0.08b | 7.59±0.08a | 7.62±0.06a | 7.59±0.08a | 7.65±0.12a |
| Day 42 | 7.14±0.08b | 7.53±0.10a | 7.62±0.06a | 7.53±0.04a | 7.57±0.12a |
| Day 56 | 7.39±0.10c | 7.54±0.04b | 7.55±0.04b | 7.55±0.02b | 7.69±0.06a |
| Day 70 | 7.45±0.06c | 7.67±0.06ab | 7.66±0.08ab | 7.61±0.08b | 7.75±0.06a |
| Day 84 | 7.36±0.06c | 7.61±0.08ab | 7.55±0.08b | 7.71±0.06a | 7.68±0.04a |
| Day 98 | 7.44±0.04b | 7.73±0.12a | 7.78±0.02a | 7.66±0.10a | 7.51±0.10b |

Note: Different lowercase letters indicated significant difference among different treatments at the same incubation stages (*P* < 0.05). Data are means ± SD, n = 4.

**Supplementary Table 5.** EC of soil porewater (mS/cm)

| Treatments  Incubation time | Control | DMAs60 | DMAs120 | DMAs120+BC1 | DMAs120+BC2 |
| --- | --- | --- | --- | --- | --- |
| Day 0 | 1.53±0.21c | 1.19±0.36c | 1.07±0.38c | 2.12±0.50b | 3.17±0.66a |
| Day 3 | 2.31±0.18c | 2.32±0.22c | 2.57±0.22c | 3.06±0.26b | 3.57±0.52a |
| Day 7 | 2.48±0.12c | 2.52±0.12c | 2.64±0.16c | 3.07±0.24b | 3.51±0.22a |
| Day 14 | 1.95±0.14c | 1.99±0.06c | 1.93±0.10c | 2.30±0.18b | 2.82±0.08a |
| Day 21 | 1.50±0.20b | 1.49±0.10b | 1.44±0.10b | 1.69±0.22b | 2.16±0.20a |
| Day 28 | 1.22±0.18c | 1.18±0.06c | 1.16±0.06c | 1.43±0.18b | 1.89±0.06a |
| Day 35 | 1.07±0.12c | 1.04±0.06c | 1.04±0.06c | 1.29±0.16b | 1.67±0.10a |
| Day 42 | 0.94±0.12c | 0.87±0.04c | 0.87±0.06c | 1.11±0.16b | 1.48±0.04a |
| Day 56 | 0.73±0.06c | 0.67±0.02c | 0.67±0.04c | 0.84±0.14b | 1.12±0.04a |
| Day 70 | 0.62±0.06c | 0.58±0.02c | 0.59±0.04c | 0.75±0.10b | 1.01±0.02a |
| Day 84 | 0.55±0.04c | 0.51±0.02c | 0.54±0.04c | 0.67±0.10b | 0.94±0.02a |
| Day 98 | 0.45±0.04c | 0.43±0.02c | 0.46±0.04c | 0.58±0.08b | 0.84±0.02a |

Note: Different lowercase letters indicated significant difference among different treatments at the same incubation stages (*P* < 0.05). Data are means ± SD, n = 4.

**Supplementary Table 6.** Fe concentration in soil porewater (mg/L)

| Treatments  Incubation time | Control | DMAs60 | DMAs120 | DMAs120+BC1 | DMAs120+BC2 |
| --- | --- | --- | --- | --- | --- |
| Day 0 | 0.37±0.14a | 0.35±0.12a | 0.39±0.14a | 0.50±0.04a | 0.45±0.10a |
| Day 3 | 20.29±2.02c | 33.17±7.36b | 53.85±3.10a | 39.20±10.34b | 41.7±5.28b |
| Day 7 | 8.95±1.34b | 7.48±0.58b | 9.26±7.22b | 3.81±0.52b | 51.06±8.41a |
| Day 14 | 27.19±6.10a | 33.04±5.10a | 27.38±5.68a | 28.32±6.94a | 35.88±13.60a |
| Day 21 | 12.39±1.24ab | 6.23±1.82c | 7.60±1.88bc | 14.95±7.42a | 14.39±3.24a |
| Day 28 | 16.79±2.04a | 9.75±1.76c | 11.94±2.04bc | 12.83±4.86abc | 15.44±2.38ab |
| Day 35 | 9.14±2.18a | 7.74±2.24a | 8.22±1.74a | 10.93±2.32a | 9.44±3.44a |
| Day 42 | 9.94±1.90a | 7.74±0.56ab | 7.60±1.34ab | 9.11±1.58ab | 6.32±2.56b |
| Day 56 | 7.38±1.94a | 5.58±1.48a | 6.48±0.74a | 7.41±1.35a | 7.97±3.32a |
| Day 70 | 6.20±1.16ab | 4.40±1.18b | 4.22±0.36b | 6.11±0.26ab | 7.32±2.58a |
| Day 84 | 6.00±1.78ab | 3.50±1.28c | 4.18±0.50bc | 5.04±1.18bc | 7.40±1.88a |
| Day 98 | 7.38±0.78a | 3.44±1.14b | 4.43±0.70b | 4.57±1.04b | 7.53±1.39a |

Note: Different lowercase letters indicated significant difference among different treatments at the same incubation stages (*P* < 0.05). Data are means ± SD, n = 4.

**Supplementary Table 7.** Mn concentration in soil porewater (mg/L)

| Treatments  Incubation time | Control | DMAs60 | DMAs120 | DMAs120+BC1 | DMAs120+BC2 |
| --- | --- | --- | --- | --- | --- |
| Day 0 | 1.11±0.44bc | 0.98±0.36bc | 0.89±0.36c | 1.71±0.52b | 2.50±0.64a |
| Day 3 | 8.96±2.68b | 10.36±1.04ab | 10.35±3.32ab | 16.02±4.10a | 13.93±5.40ab |
| Day 7 | 1.82±0.68cd | 4.11±2.06b | 3.43±1.32bc | 1.27±0.68d | 12.29±1.20a |
| Day 14 | 5.05±1.22a | 6.18±1.78a | 5.48±2.32a | 7.12±1.42a | 7.52±2.24a |
| Day 21 | 3.53±0.36b | 4.36±0.30ab | 2.95±1.14b | 4.53±1.42ab | 6.57±0.36a |
| Day 28 | 3.63±0.18b | 2.70±0.58c | 3.54±0.52bc | 4.50±0.70a | 5.03±0.62a |
| Day 35 | 3.23±0.68a | 2.71±0.38a | 3.17±0.28a | 3.51±0.30a | 3.62±1.06a |
| Day 42 | 3.09±0.30bc | 2.79±0.22c | 2.75±0.34c | 3.35±0.26b | 3.85±0.32a |
| Day 56 | 2.28±0.26a | 2.01±0.06a | 2.08±0.22a | 2.67±0.90a | 2.74±0.76a |
| Day 70 | 1.88±0.38bc | 1.74±0.08c | 1.71±0.24c | 2.19±0.18b | 2.77±0.24a |
| Day 84 | 1.69±0.24b | 1.59±0.02b | 1.65±0.22b | 1.98±0.28b | 2.61±0.32a |
| Day 98 | 1.55±0.18bc | 1.47±0.10c | 1.53±0.20bc | 1.87±0.32ab | 2.08±0.34a |

Note: Different lowercase letters indicated significant difference among different treatments at the same incubation stages (*P* < 0.05). Data are means ± SD, n = 4.

**Supplementary Table 8.** Al concentration in soil porewater (μg/L)

| Treatments  Incubation time | Control | DMAs60 | DMAs120 | DMAs120+BC1 | DMAs120+BC2 |
| --- | --- | --- | --- | --- | --- |
| Day 0 | 386.39±71.36a | 388.74±44.48a | 428.27±32.60a | 427.56±14.94a | 373.44±54.88a |
| Day 3 | 335.51±37.50b | 361.60±32.78ab | 341.94±32.16b | 402.27±17.78a | 350.45±25.14b |
| Day 7 | 384.34±28.08a | 400.79±15.22a | 374.01±51.86a | 402.08±40.78a | 369.27±23.58a |
| Day 14 | 378.69±11.06ab | 379.40±13.32ab | 372.39±14.86b | 405.08±19.28a | 401.85±24.36a |
| Day 21 | 411.12±8.68a | 424.86±24.16a | 412.31±22.40a | 445.84±95.34a | 410.54±12.66a |
| Day 28 | 414.68±11.56a | 419.10±24.04a | 405.00±18.00a | 408.50±24.46a | 406.75±15.90a |
| Day 35 | 413.33±10.38ab | 401.38±17.14abc | 380.68±27.80c | 432.32±23.94a | 390.27±15.18bc |
| Day 42 | 396.00±36.96a | 403.64±45.54a | 392.29±35.50a | 392.51±11.80a | 406.95±36.42a |
| Day 56 | 542.99±49.38ab | 549.43±55.32a | 520.55±13.68ab | 515.40±23.14ab | 485.09±26.10b |
| Day 70 | 531.26±14.38ab | 528.17±36.18ab | 513.53±37.92b | 531.64±18.62ab | 599.98±97.48a |
| Day 84 | 518.56±11.08a | 526.63±11.84a | 543.73±56.12a | 527.76±45.20a | 533.05±37.20a |
| Day 98 | 499.40±12.34a | 505.80±6.48a | 506.40±6.88a | 508.89±34.54a | 500.23±33.38a |

Note: Different lowercase letters indicated significant difference among different treatments at the same incubation stages (*P* < 0.05). Data are means ± SD, n = 4.
